# Self-administered acupressure for allergic rhinitis: study protocol for a randomized, single-blind, non-specific controlled, parallel trial

**DOI:** 10.1186/s13063-019-3495-0

**Published:** 2019-06-25

**Authors:** Yaqun Liang, George Binh Lenon, Angela Wei Hong Yang

**Affiliations:** Discipline of Chinese Medicine, School of Health and Biomedical Sciences, RMIT University, PO Box 71, Bundoora, VIC 3083 Australia

**Keywords:** Hay fever, Allergic disease, Acupuncture, Self-massage, Evidence-based Chinese medicine

## Abstract

**Background:**

Allergic rhinitis (AR) is an IgE-mediated inflammatory disease. Current conventional therapies for AR are unsatisfactory. Acupuncture has been recommended as an optional treatment for AR patients who are interested in non-pharmacotherapy in the new clinical practice guidelines for AR. Acupressure is a sub-type of acupuncture which is non-invasive with a low risk and can be self-administered. However, the current limited evidence is compromised by the high risk of bias and heterogeneity of methodology. Therefore, rigorously designed randomized controlled trials (RCTs) are needed. This proposed RCT aims to evaluate the efficacy and safety of self-administered acupressure for the management of AR.

**Methods/design:**

We have designed a randomized, single-blind, non-specific controlled, two-arm, parallel clinical trial involving a 2-week run-in period, a 4-week intervention period and an 8-week follow-up period. The eligible participants will be randomized into either a specific or a non-specific acupressure group. They will be required to perform self-administered acupressure on either five specific acupressure points or five non-specific acupressure points, 1 min for each point, twice a day for 4 weeks. Participants will be asked to complete self-administered questionnaires for outcome measures including a 7-point scale of symptom severity, the Rhinoconjunctivitis Quality of Life Questionnaire with Standardized Activities (RQLQs), relief medication scores, adverse events and participants’ opinion of this study at the different assessment points throughout the trial period. Data will be analyzed by the chi-square or *t* test using Statistical Package for Social Science (SPSS) software.

**Discussion:**

The findings from this study should provide scientific evidence for the efficacy and safety of self-administered acupressure for the management of AR. This study may assist the development of a non-cost, non-invasive self-management method for AR sufferers.

**Trial registration:**

Australian and New Zealand Clinical Trials Registry (ANZCTR), ID: ACTRN12617001106325 Registered on 28 July 2017.

**Electronic supplementary material:**

The online version of this article (10.1186/s13063-019-3495-0) contains supplementary material, which is available to authorized users.

## Background

Allergic rhinitis (AR) is an immunoglobulin E (IgE)-mediated immunologic inflammatory response of the nasal membrane after exposure to certain allergens which include pollens, dust mites, moulds, smoke, animal dander, air pollutants and occupational agents [1–3]. Patients with AR present with nasal symptoms such as sneezing, nasal itching, nasal congestion and a runny nose; which may be combined with non-nasal symptoms such as itchy and watery eyes, as well as an itchy and sore throat [2, 4]. AR has a high prevalence worldwide ranging from 10 to 30% of the population affected [5]. In Australia, AR is a common respiratory condition that affects approximately 20% of population and the cost of medication from the pharmacy suppliers increased from $107.8 million to $226.8 million between 2001 and 2010 [6, 7] . The troublesome nature of AR symptoms have a significant impact on patients’ quality of life such as work and school performance, sleep and mental health [2]. Moreover, AR has a high impact on asthma and is associated with rhinosinusitis and other comorbidities such as conjunctivitis and chronic cough [2, 6, 8].

Current management of AR includes allergen avoidance, pharmacotherapies (such as H_1_-antihistamines, corticosteroids, chromones, decongestants, anti-cholinergic drugs and anti-leukotrienes) and allergen-specific immunotherapy [2]. Most of the medications for AR are over-the-counter in Australia [6]. However, these medications are unable to provide full symptomatic relief and some of them are associated with undesirable side effects such as drowsiness and dryness in the nasal cavity. Systemic glucocorticosteroids cannot be used in the long term due to systemic side effects, such as suppressed immune system, osteoporosis, hypertension, diabetes, and especially impact on children’s growth [2, 3]. Those could be the major reasons for more and more patients with AR seeking help from complementary and alternative medicine including acupuncture [9].

Acupuncture has a long history for the management of AR. Currently, it has been recommended as an optional treatment for AR patients who are interested in non-pharmaceutical therapies in the Clinical Practice Guidelines for AR developed by the American Academy of Otolaryngology – Head and Neck Surgery and endorsed by the American Academy of Family Physicians [10]. Acupressure is a sub-type of acupuncture. It is a non-invasive therapeutic method applying physical pressure to certain acupuncture points by finger, elbow, hand or with various devices to treat diseases [11]. The popularity of acupressure has increased over recent years and has been the fourth preferred complementary and alternative therapy in hospitalized patients in Australia [12]. Several clinical studies have validated the effects of acupressure for various health conditions; for instance, nausea, vomiting and pain after caesarean delivery [13], hypertension [14] and constipation [15]. A systematic review of randomized controlled trials (RCTs) in acupressure for respiratory allergic diseases indicated that acupressure is safe for symptomatic relief of AR and asthma. However, no reliable evidence of efficacy could be identified due to the small number of included RCTs, the heterogeneity of the study design and a high/unclear risk of bias [16]. There is a need to perform rigorously designed RCTs to investigate the efficacy and safety of acupressure for the management of AR.

This study aims to investigate the efficacy and safety of self-administered acupressure on symptomatic relief and health-related quality of life improvement in adults with AR by conducting a superiority RCT. The study will also explore whether self-administered acupressure is effective in reducing the usage of Western medications in the management of AR.

## Materials/design

### Trial design

This study will be conducted at the Chinese Medicine Clinical Trial Laboratory in Royal Melbourne Institute of Technology (RMIT) University, Bundoora West and City campuses. It has been designed as a randomized, single-blind, non-specific controlled (i.e., using true acupuncture points without specific indications for AR in the control group), and two-arm parallel clinical trial according to the Guideline of National Statement on Ethical Conduct in Human Research 2007 (Updated May 2018) [17]. The trial will consist of a 2-week run-in period, a 4-week intervention period and an 8-week follow-up period. This study protocol was developed as required by the Standard Protocol Items: Recommendations for Interventional Trials (SPIRIT) (Additional file 1) [18] and the Revised STandards for Reporting Interventions in Clinical Trials of Acupuncture (STRICTA): extending the Consolidated Standards of Reporting Trials (CONSORT) Statement [19].

Ethics approval has been obtained from RMIT Human Research Ethics Committee (HREC) (project number: 20742). The RMIT HREC will oversee the conduct of this trial. The trial has been registered with the Australian and New Zealand Clinical Trials Registry (ACTRN12617001106325) (Additional file 2).

### Participants

The participants will be taken from the general population in Melbourne, Australia and recruited through advertisements on the Internet, posters, notice boards, leaflets, media release, social media, local newspapers, and newsletters. Participation in this research project is voluntary. The participants will be selected according to the inclusion and exclusion criteria listed in Table 1 below.

**Table 1:** Inclusion and exclusion criteria of participants

Inclusion criteria	Exclusion criteria
• Aged ≥ 18 years • ≥ 2-year history of AR • (+) skin-prick test • Currently not involved in other clinical trials • Agree to make themselves available for the period of the study • Provide written consent for participation • Will not travel overseas or interstate for 14 weeks of the trial period • Have access to a computer and the Internet	• Current systemic corticosteroid therapy • Other current active respiratory disease (e.g., asthma) • Have used acupuncture/acupressure for respiratory or allergic diseases within the last month • Structural defects of the upper respiratory tract • Practitioner of Chinese medicine, an acupuncturist or a practitioner of Chinese medicine • Pregnancy • Travel overseas or interstate in the 14 weeks of the trial period • History of HIV, hepatitis B or C • Nasal polyposis • Unable to read and write English

*AR* allergic rhinitis

### Sample size

The sample size was calculated using the program G*Power 3.1.9 [20]. Due to the absence of a similar acupressure study, the sample size estimation was based on an acupuncture study [21] that is closest to our study as we feel that using the acupuncture study would produce the best possible estimates of effect size and sample size. The effect size estimate in this acupuncture study was based on the main outcome variable (i.e., total nasal symptom score) which is also used in our study. We have also inflated the estimated sample size to cater for a 10% dropout which normally occurs in studies involving acupuncture. The effect size estimate is 0.6505. To achieve 80% power at the significance level 0.05 we need 45 subjects per group taking into consideration a 10% dropout rate.

### Recruitment

**Fig. 1:**
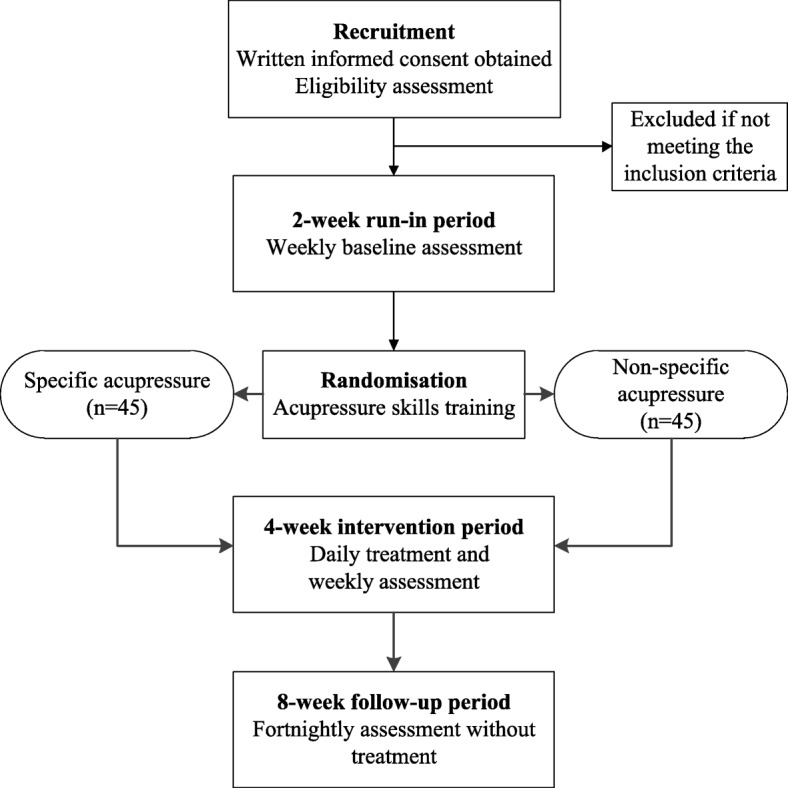
Flow chart of the clinical trial procedures

People who are interested in taking part in this trial will be provided with a Participant Information Sheet with Consent Form (PIS-CF) and asked to complete a general questionnaire and screening questionnaire if they agree with the PIS-CF. Written consent will be obtained from each participant by the investigators before an initial interview. The activities in the initial interview will include the allergen skin-prick test, a physical examination and a Chinese medicine differential diagnosis [22] to achieve an AR diagnosis. The participants meeting the inclusion criteria will enter the 2-week run-in period. During this period, every participant will be asked to conduct two sets of weekly baseline assessment including a 7-point scale symptom severity assessment form [23], a Rhinoconjunctivitis Quality of Life Questionnaire with Standardized Activities (RQLQs) [24] and a medication usage form. All baseline assessment forms will be submitted in the first intervention session. Figure 1 outlines the procedures of the clinical trial.

### Randomization and blinding

To minimize bias, eligible participants will be randomized into either the specific acupressure treatment group or the non-specific acupressure control group with an allocation ratio of 1:1. Randomization will be conducted after baseline assessment using a computer program run by an independent researcher who is not directly involved in the trial. The randomization codes will be put into individually sealed opaque envelopes with sequel trial numbers. The sealed opaque envelopes contain the information on the location of specific or non-specific acupressure points for self-administered acupressure. Each participant will be asked to pick one sealed envelope from the pack of all the envelopes and pass it onto the registered acupuncturist. This acupuncturist is the only person who knows the participants’ group allocation in the trial. Neither the participants nor other investigators (such as data entry personnel and data analyst) will know the participants’ allocation to receive specific or non-specific acupressure treatment. The randomization codes and the allocation of the participants will be revealed once the RCT is completed. However, in the event of the need of this grouping information (e.g., a participant experiencing severe adverse events), the investigators will access the grouping data on the request of the physician. The relevant information will be documented in the participant’s case report form.

### Interventions

**Fig. 2:**
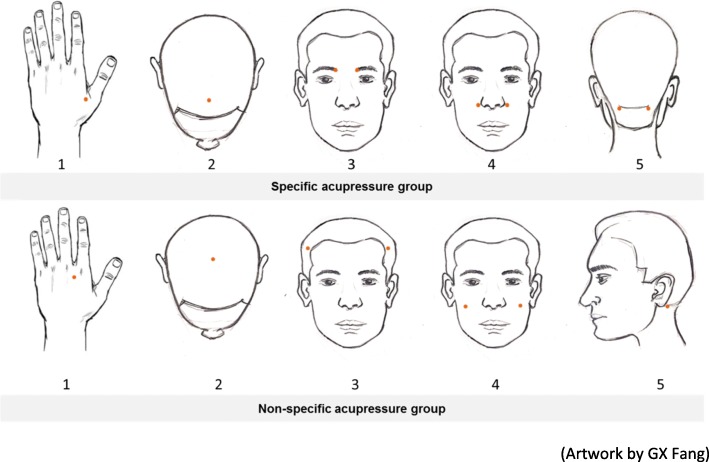
The location of five specific and five non-specific acupressure points

After randomization, the registered acupuncturist will provide detailed instructions and training on self-administered acupressure techniques to each participant individually. A pictorial instruction showing the location of acupressure points will be provided to the participants for further reference. Participants will be asked to perform self-administered acupressure on either five specific or non-specific acupressure points following the sequence from point 1 to point 5. The points will be self-pressed with maximal tolerable pressure but still within the comfortable range for 1 min each point, twice a day for 4weeks and complete the self-assessment on a weekly basis. During the treatment period, participants are required to have a weekly visit to the Clinical Trial Laboratory and the registered acupuncturist will monitor their skills to ensure the accurate performance of self-administered acupressure techniques. Messages will be sent to each participant to remind them to continue performing acupressure and attend the Clinical Trial Laboratory during each week. Figure 2 below illustrates the location of five specific acupressure points and five non-specific acupressure points.

#### Specific self-administered acupressure

Participants in the specific acupressure group will apply self-administered acupressure on five specific acupressure points including LI4 *Hegu*, GV23 *Shangxing*, BL2 *Zanzhu*, LI20 *Yingxiang* and GB20 *Fengchi*. The acupressure points were selected based on a review of the classical and modern literature [11, 16, 22, 25].

#### Non-specific self-administered acupressure

Participants in the non-specific acupressure group will apply self-administered acupressure on another five non-specific acupressure points which are true acupuncture points on the body but not specifically indicated for AR treatment according to literature including *Extra Luozhen*, GV20 *Baihui*, GB4 *Hanyan*, SI18 *Quanliao* and GB12 *Wangu* [11, 25].

#### Symptomatic relief medications

Participants will be allowed to continue their existing management for AR, such as pharmacological therapy. They will be asked to record the details of all medications used in the medication usage form.

### Outcome measures

**Fig. 3:**
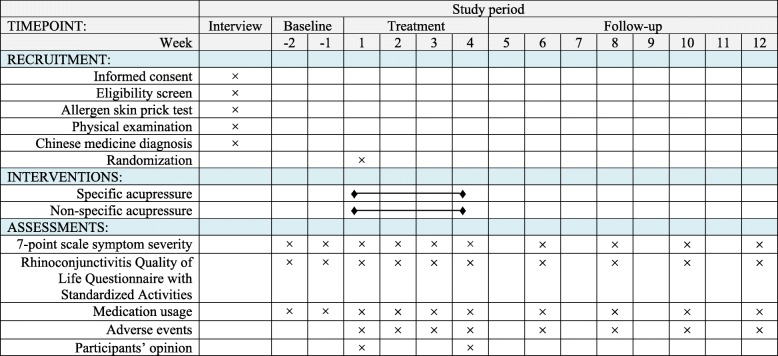
The schedule of recruitment, interventions and assessments

Primary and secondary outcome measures will be included in this clinical trial. The comparison between two groups will be at baseline, at the end of the treatment period and at the end of the follow-up period. The RCT schedule of recruitment, interventions and assessments is shown in Fig. 3.

#### Primary outcome measures

The primary outcome measure will be AR symptomatic relief measured by a 7-point scale of symptom severity with the score ranging from 1 to 7. It contains four domains including I (nasal symptom severity), II (non-nasal symptom severity) and IV (quality-of-life assessment of rhinitis severity) where a higher score indicates more severe symptoms and better quality of life (QoL) as well as III (global assessment of nasal and non-nasal symptom severity) where a lower score indicates more severe symptoms [23]. The scores of four individual domains will be assessed separately at baseline and during the intervention period on a weekly basis and in the follow-up period on a fortnightly basis. The primary outcome of the intervention will be a combination of the four domains.

#### Secondary outcome measures

The secondary outcome measures will consist of the RQLQs [24], relief medication scores, adverse events and participants’ opinion about this clinical trial. The RQLQs is used to assess the patient’s quality of life and daily activities which contain seven domains with scores from 0 to 6; a higher score indicates a more severe impact on QoL [24]. The use of anti-allergic medication is calculated as each daily dose of antihistamines or decongestant nasal spray or eye drops was equivalent to 1 point; orally administered antihistamines as 2 points and a steroid nasal spray or eye drops as 3 points [26]. If the participants use orally administered corticosteroids or acupuncture for AR during the trial period, they will be asked to discontinue the self-administered acupressure treatment and considered as withdrawing from this study. Adverse events will be self-monitored and recorded in the adverse events form. If there are severe adverse events from the self-administered acupressure, the relevant participants will be asked to terminate the study and contact the researchers immediately. They will be referred to general practitioners or the emergency department for management. All severe adverse events will be investigated and reported in writing to the RMIT HREC immediately. A questionnaire will be used to seek participants’ opinion on the expectancy of self-administered acupressure for AR and the credibility of the blinding method used in the study.

RQLQs and relief medication scores will be assessed at baseline, intervention period (weekly) and follow-up period (fortnightly). Acupressure dosage and adverse events will be recorded in a weekly form during the treatment period to monitor participants’ compliance and safety, and adverse events will be further assessed fortnightly in the follow-up period. Participants’ opinion will be assessed at the end of first and final weeks of the intervention period.

### Data management

An individual file for each participant will be used to archive a hard copy of the case record forms including informed consent, results of the allergen skin-prick test, results of the physical examinations and all completed questionnaires. The files will be stored in the lock-up cabinet. The electronic submission documents will be kept in a RMIT University password-protected computer. Only the investigators of this study will have the authority to access the data. The results of the study and the grouping information of the participants will be provided to the individual after completion of the trial. Publications will only report aggregated data. Personal identity will not be disclosed.

### Data analysis

Statistical analyses will be performed by an independent statistician at RMIT University using IBM SPSS Statistics for Windows Version 25 software (IBM Corp., Armonk, NY, USA). The chi-square test will be used to assess differences between the two treatment groups on categorical variables such as characteristic and demographic data at baseline. The *t* test will be used to assess differences between the two treatment groups on the primary outcomes (nasal and non-nasal symptom scores, QoL, global assessment score as well as the total score of the four domains) and secondary outcome variables (RQLQs and relief medication scores) at the end of treatment and follow-up periods. All analyses will follow the intention-to-treat principle. The Worst-Case-Scenario method will be employed to deal with the missing data. That is, we will carry forward the worst possible outcomes for the missing data. Participants enrolled in the study with data from at least one treatment will be included for analysis. No interim analysis will be performed due to the short duration of the trial.

All comparisons will be two-tailed and *p* values < 0.05 will be considered as statistically significant. Subgroup analysis may be performed according to syndrome differential diagnosis of Chinese medicine, severity of symptoms or age group of participants.

## Discussion

Acupuncture has been recommended as an alternative treatment for AR in the new Clinical Practice Guidelines [10], based on existing evidence from high-quality RCTs, with positive effects [21, 26, 27]. Acupressure is a sub-type of acupuncture which may have a similar mechanisms of action to acupuncture; that is, stimulation of acupuncture points could activate the fiber terminals of peripheral nerves, trigger neurological responses [28] and decrease inflammatory cytokine and neuropeptide levels [29]. However, the clinical effects and safety of acupressure have not been fully determined. A recent systematic review has identified the need for rigorously designed RCTs of acupressure for AR due to weaknesses of current limited RCTs (e.g., significant heterogeneity and high/unclear risk of bias) [16]. This proposed RCT aims to fill in this knowledge gap by addressing all the weaknesses identified in this review.

This RCT has been rigorously designed as a randomized, single-blind, non-specific controlled clinical trial to minimize risks of bias. An independent statistician will generate randomization numbers using computer software and the randomization number will be assigned to participants using sealed opaque envelops. This procedure ensures adequate randomization and allocation concealment of participants. Due to the nature of acupressure, blinding of the acupuncturist is impossible for this trial. However, participants will be blinded during the entire randomization and intervention processes. Selection of acupressure points in treatment and control groups can be challenging as the size of fingers limits the chance of choosing the non-actual points 1 to 3 cm next to the real points which is the most popularly used sham control method in acupuncture/acupressure RCTs [30, 31]. This RCT adopts specific and non-specific points which are in the same area but 3 to 4 cm in between in the treatment and control groups which aims to maximizes the possibility of achieving successful blinding of participants [32]. In addition, all the interventions and the outcome measures will be administered by participants themselves, which avoids involving third-party assessors to reduce potential performance bias since blinding of assessors is considered more important than blinding practitioners since blinded assessments are less likely to bring biases to subjective outcomes [33]. When dealing with missing data, we will utilize the Worst-Case-Scenario method and intention-to-treat approach to manage the attrition bias. It is ideal to employ objective measures such as serum allergen-specific IgE to further investigate the mechanisms of action of acupressure for AR management. However, it is not feasible in this proposed RCT due to limited funding.

In summary, this protocol offers a standard regimen to guide the conduct of a RCT. By performing the rigorously designed RCT, this research project should make a significant contribution to the literature on self-administered acupressure. It may lead to the adoption of self-administered acupressure as a low-risk, non-invasive, non-pharmaceutical intervention for AR and could also assist the self-management of AR.

### Trial status

This protocol is version 1. The advertisement for the recruitment began in August 2017 after the ethics approval. It is anticipated that the recruitment will be completed by the end of June 2020. The recruitment is currently in progress.

## Additional files

Additional file 1: Standard Protocol Items: Recommendations for Interventional Trials (SPIRIT) 2013 Checklist: recommended items to address in a clinical trial protocol and related documents. (DOC 124 kb)

Additional file 2: Trial registration with the Australian and New Zealand Clinical Trials Registry (ANZCTR) (PDF 176 kb)

Additional file 3: Informed consent materials. (DOCX 58 kb)

## Data Availability

Not applicable. This paper is a protocol for a randomized clinical trial and does not contain any data.

## Abbreviations

ANZCTR: Australian and New Zealand Clinical Trials Registry
AR: Allergic rhinitis
CONSORT: Consolidated Standards of Reporting Trials
HREC: Human Research Ethics Committee
IgE: Immunoglobulin E
PIS-CF: Participant Information Sheet with Consent Form
QoL: Quality of life
RCT: Randomized controlled trial
RQLQs: Rhinoconjunctivitis Quality of Life Questionnaire with Standardized Activities
SPIRIT: Standard Protocol Items: Recommendations for Interventional Trials
SPSS: Statistical Package for Social Science
STRICTA: STandards for Reporting Interventions in Clinical Trials of Acupuncture
