# Supplementary material for: Self-administered acupressure for allergic rhinitis: study protocol for a randomized, single-blind, non-specific controlled, parallel trial
Source: Trials. 2019 Jun 25;20:382. doi: 10.1186/s13063-019-3495-0 (PMC6593608; doi:10.1186/s13063-019-3495-0)
Supplement: Supplementary file 2 — Trial registration with the Australian and New Zealand Clinical Trials Registry (ANZCTR) (PDF 176 kb) [file 13063_2019_3495_MOESM2_ESM.pdf]

LOG OFF Logged in as Yaqun Liang
[Account details]

DEFINITIONS HINTS AND TIPS FAQs REGISTER TRIAL MY TRIALS

Register a trial

Acknowledgment	Step 1: Titles & IDs	Step 2: Health condition	Step 3: Intervention/exposure
Step 4: Outcomes	Step 5: Eligibility	Step 6: Study design	Step 7: Recruitment
Step 8: Funding & Sponsors	Step 9: Ethics & Summary	Step 10: Contacts	Review & Submit

**Request number**	373370
**Current page**	Review

< BACK

Trial registered on ANZCTR

**Trial ID**	ACTRN12617001106325
**Ethics application status**	Approved
**Date submitted**	25/07/2017
**Date registered**	28/07/2017
**Type of registration**	Prospectively registered

Titles & IDs

**Public title**	Self-administered acupressure for the management of allergic rhinitis
**Scientific title**	Effects of self-administered specific acupressure versus non-specific acupressure for the management of allergic rhinitis
**Secondary ID [1]**	Nil
**Universal Trial Number (UTN)**	U1111-1199-8020
**Trial acronym**	ARCT
**Linked study record**	

Health condition

Health condition(s) or problem(s) studied:

Allergic rhinitis

Condition category	Condition code
Alternative and Complementary Medicine	Other alternative and complementary medicine
Inflammatory and Immune System	Allergies
Respiratory	Other respiratory disorders / diseases

Intervention/exposure

**Study type**	Interventional
**Description of intervention(s) / exposure**	Participants will complete self-administered questionnaires for the severity of symptoms, quality of life and medication use during the 2-week run-in period. After randomisation at the first visit to the clinic laboratory during the treatment period, the registered acupuncturist will provide detailed instructions to each

participant in a 15-minute session on a one-on-one basis. Each participant will also receive an information sheet ("Location of 5 Acupoints for Self-administered Acupressure") showing the details of the location of acupuncture points and administration.

Participants in specific acupressure group will perform self-administered acupressure to five specific acupuncture points bilaterally, including Hegu (LI 4), Shangxing (GV 23), Zanzhu (BL 2), Yingxiang (LI 20) and Fengchi (GB 20). Participants will apply pressure to each acupuncture point for one minute, twice a day for four consecutive weeks. Participants will be asked to have a 10-minute one-on-one weekly visit for the four-week intervention period. The same acupuncturist will reinforce the participants' self-administered acupressure skills in every visit during the treatment period and ensure their techniques are correct.

Intervention code [1] Treatment: Other

Comparator / control treatment Participants will complete self-administered questionnaires for the severity of symptoms, quality of life and medication use during the 2-week run-in period. After randomisation at the first visit to the clinic laboratory during the treatment period, the registered acupuncturist will provide detailed instructions to each participant in a 15-minute session on a one-on-one basis. Each participant will also receive an information sheet ("Location of 5 Acupoints for Self-administered Acupressure") showing the details of the location of acupuncture points and administration.

Participants in the control group will perform self-administered acupressure on five non-specific acupuncture points, including Extra-Luozhen, Baihui (GV 20), Hanyan (GB 4), Quanliao (SI 18) and Wangu (GB 12). Participants will apply pressure to each acupuncture point for one minute, twice a day for four consecutive weeks. Participants will be asked to have a 10-minute one-on-one weekly visit for the four-week intervention period. The same acupuncturist will reinforce the participants' self-administered acupressure skills in every visit during the treatment period and ensure their techniques are correct.

Control group Placebo

Outcomes

Primary outcome [1] Symptom scores assessed by 7-point scale questionnaire in the severity of symptoms

Timepoint [1] Baseline, end of treatment (4 weeks) and end of follow-up period (8 weeks)

Secondary outcome [1] Quality of life assessed by Rhinoconjuctivitis Quality of Life Questionnaire with Standardised Activities (RQLQs)

Timepoint [1] Baseline, end of treatment (4 weeks) and end of follow-up period (8 weeks)

Secondary outcome [2] Medication usage (name, dose and frequency of Western medication used for managing allergic rhinitis symptoms) recorded in the Medication use form

Timepoint [2] Baseline, end of treatment (4 weeks) and end of follow-up period (8 weeks)

Secondary outcome [3] Adverse events of self-administered acupressure recorded in adverse event form (eg. pain in the acupuncture point regions)

Timepoint [3] During treatment period (4 weeks) and follow-up period (8 weeks)

Secondary outcome [4] Participants' opinion about self-administered acupressure by Participants' Opinion of Self-administered Acupressure Credibility Expectancy Questionnaire (9-point scale)

Timepoint [4] At the first week and the final week of the 4-week treatment period

Eligibility

Key inclusion criteria

• Aged 18 years old and above;
• A history of at least two years of typical symptoms of allergic rhinitis;
• Have a positive skin prick test to one or more of the allergens such as Seven-grass mix, Perennial Rye, Ragweed, House mite, Animal's dander or Mould;
• Currently not involved in other clinical trials for the treatment of allergic rhinitis;
• Provide written consent for participant to sign;
• Have access to computer and internet; and
• Will not travel overseas or interstates for the 14 weeks of trial period.

Minimum age 18 Years

Maximum age No limit

Gender Both males and females

Can healthy volunteers participate? No

Key exclusion criteria

• Current systemic corticosteroid therapy;
• Other current active respiratory disease such as asthma;
• Nasal polyposis;
• Other structural defects of the upper respiratory tract;
• History of asthma, HIV, Hepatitis B or C;
• Current pregnancy;
• Have used acupuncture/acupressure for respiratory or allergic diseases within the last month;
• Chinese herbal medicine practitioner, acupuncturist, past or current Chinese medicine student;
• Travel overseas or interstates in the 14 weeks of trial period; or
• Do not understand English.

Study design

Purpose of the study
Treatment

Allocation to intervention
Randomised controlled trial

Procedure for enrolling a subject and allocating the treatment (allocation concealment procedures)
Each computer generated random number will be kept in a sealed opaque envelope until the participant is randomised prior to the first treatment.

Methods used to generate the sequence in which subjects will be randomised (sequence generation)
Randomisation sequence will be computer generated by an independent researcher.

Masking / blinding
Blinded (masking used)

Who is / are masked / blinded?
The people receiving the treatment/s

The people assessing the outcomes
The people analysing the results/data

Intervention assignment
Parallel

Other design features
The grouping information will only be known to the registered acupuncturist who will provide instructions to the participants. This acupuncturist will not disclose the grouping information to the participants or other trial investigators.

Phase
Not Applicable

Type of endpoint(s)
Safety/efficacy

Statistical methods / analysis
Data analysis will be conducted using the Statistical Package for Social Science (SPSS).

Recruitment

Recruitment status
Not yet recruiting

Update
Recruiting

Reason
The clinical trial has started to recruit participants since 1st August 2017.

Date of first participant enrolment

Anticipated 31/07/2017

Actual

Update 1/11/2017

Reason The first participant came in for interview.

Date of last participant enrolment

Anticipated

Actual

Date of last data collection

Anticipated

Actual

Sample size

Target 110

Accrual to date

Update 15

Reason 15 participants enrolled in the pilot study,

Final

Recruitment in Australia

Recruitment state(s)
VIC

Funding & Sponsors

Funding source category [1]
University

Name [1]
RMIT University

Address [1]
School of Health and Biomedical Sciences
RMIT University
PO Box 71
Bundoora VIC 3083

Country [1]
Australia

Primary sponsor type
University

**Name**	RMIT University
**Address**	School of Health and Biomedical Sciences<br>RMIT University<br>PO Box 71<br>Bundoora VIC 3083
**Country**	Australia
**Secondary sponsor category [1]**	None
**Name [1]**	
**Address [1]**	
**Country [1]**	

Ethics approval

**Ethics application status**	Approved
**Ethics committee name [1]**	RMIT University Human Research Ethics Committee
**Ethics committee address [1]**	Research and Innovation office<br>GPO Box 2476<br>MELBOURNE VIC 3001
**Ethics committee country [1]**	Australia
**Date submitted for ethics approval [1]**	
**Approval date [1]**	02/06/2017
**Ethics approval number [1]**	20742

Summary

**Brief summary**	This randomised controlled trial aims to investigate the efficacy and safety of self-administered acupressure for the management of allergic rhinitis. Acupressure is a subtype of acupuncture without needle insertion. It applies fingers to press points on the body. The study consists of 2-week run-in, 4-week treatment and 8-week follow-up period.
**Trial website**	
**Trial related presentations / publications**	
**Public notes**	
**Private notes**	

Contacts

Principal investigator

**Title**	Dr
**Name**	Angela Yang
**Address**	School of Health and Biomedical Sciences<br>RMIT University<br>PO Box 71<br>Bundoora VIC 3083
**Country**	Australia
**Phone**	+61 3 9925 7175
Fax	
**Email**	angela.yang@rmit.edu.au

Contact person for public queries

**Title**	Dr
**Name**	Angela Yang
**Address**	School of Health and Biomedical Sciences<br>RMIT University<br>PO Box 71<br>Bundoora VIC 3083
**Country**	Australia
**Phone**	+61 3 9925 7175
Fax	

Email angela.yang@rmit.edu.au

Contact person for scientific queries

Title Dr

Name Angela Yang

Address School of Health and Biomedical Sciences
RMIT University
PO Box 71
Bundoora VIC 3083

Country Australia

Phone +61 3 9925 7175

Fax

Email angela.yang@rmit.edu.au

Contact person responsible for updating information

Title

Name

Address

Country

Phone

Fax

Email

< BACK

ANZCTR

Home
About us
Statistics
Useful links
News
Contact
Privacy
Terms and conditions

Register a trial

Create account
Login
How to register a trial
How to update a trial
Data item definitions
Hints and tips
FAQs

Search for a trial

Find a trial
How to search
How to get involved

Major funders

Privacy | Disclaimer
Web design by G Squared

Copyright © Australian New Zealand Clinical Trials Registry. All rights reserved.
