# Supplementary material for: Self-administered acupressure for allergic rhinitis: study protocol for a randomized, single-blind, non-specific controlled, parallel trial
Source: Trials. 2019 Jun 25;20:382. doi: 10.1186/s13063-019-3495-0 (PMC6593608; doi:10.1186/s13063-019-3495-0)
Supplement: Supplementary file 3 — Informed consent materials. (DOCX 58 kb) [file 13063_2019_3495_MOESM3_ESM.docx]

**
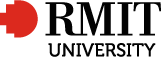
**

**School of Health and Biomedical Sciences**

**Participant Information Sheet/Consent Form**

| **Title** | Self-administered acupressure for the management of allergic rhinitis: Randomised controlled trials |
| --- | --- |
| **Chief Investigator/Senior Supervisor** | Dr Angela Wei Hong Yang |
| **Associate Investigator(s)/Associate Supervisor(s)** | Dr George Binh Lenon |
| **Principal Research Student(s)** | Ms Yaqun Liang |

**What does my participation involve?**

**1 Introduction**

You are invited to take part in this research project, which is called Self-administered acupressure for the management of allergic rhinitis: Randomised controlled trials. You have been invited because you have this medical condition and may meet the inclusion criteria of our clinical trial.

This Participant Information Sheet/Consent Form informs you about the research project and explains the processes involved. Knowing what is involved will help you decide if you want to participate in the research.

Please read this information carefully. Ask questions about anything that you don’t understand or want to know more about. Before deciding whether or not to take part, you might want to discuss this with a relative or friend.

Participation in this research is voluntary. If you don’t wish to take part, you don’t have to.

If you decide you want to take part in the research project, you will be asked to sign the consent section. By signing it you are telling us that you:

• Understand what you have read

• Consent to take part in the research project

You will be given a copy of this Participant Information Sheet and Consent Form to keep.

**2 What is the purpose of this research?**

The prevalence of allergic rhinitis (hay fever) has increased worldwide in the last decades. Acupuncture has been used for hay fever management for many years and acupuncture has been considered as one option for the management of hay fever. However, acupuncture is associated with out-of-pocket expenses as it is not covered by the national Medicare scheme, and some patients do not take acupuncture due to fear of needles.

Acupressure is a sub-type of acupuncture, it is a non-invasive technique using fingers to press points to achieve the therapeutic effects and it can be self-administered. The Discipline of Chinese Medicine at RMIT University has extensive experience in research into hay fever, and we now would like to further investigate whether acupressure could relieve hay fever symptoms, decrease the usage of medications and improve the quality of life for hay fever sufferers. The relevant information (such as questionnaires) will be provided to participants via internet and/or during visits. It is anticipated that this non-invasive, non-cost therapeutic method can be widely used in the Australian community which will further reduce the financial burden of our health care system.

The results of this research will be used by the researcher, Ms Yaqun Liang, to obtain a Doctor of Philosophy degree in complementary medicine.

This research has been initiated by the researcher, Dr Angela Wei Hong Yang, Discipline of Chinese Medicine, RMIT University.

**3 What does participation in this research involve?**

If you would like to participate in this research project, you must be 18 years old or above and have had hay fever for at least two years. You and your condition meet the inclusion criteria of this study. You should stay at the same address or in the same suburb for the entire 14 weeks since hay fever is highly related to the environment and allergens can be different in different areas. The change of the location can influence the results of the research project. You will not be able to participate if you are currently under systemic corticosteroid therapy, or having a current active respiratory disease such as asthma, or have received acupuncture or acupressure treatment within the last month, or will travel overseas or interstates during the trial period, or acupuncturist, Chinese herbal medicine practitioner, current or past Chinese medicine students. We will need to know your medical history in relation to allergies, respiratory diseases and other conditions relevant to hay fever.

If you decide to take part in the research project, you will first be asked to complete two enclosed questionnaires (General Information Questionnaire and Screening Questionnaire for the medical history of hay fever) online. These will help determine if you are eligible to take part. Completing the questionnaires will take approximately 20-30 minutes.

The screening questionnaire may show that you cannot participate in the research project. If you do not meet the inclusion criteria, you will be informed by email within two weeks of receiving your submission.

If the screening questionnaire shows that you meet the requirements, and then you will be invited to the Clinical Trial Laboratory for an assessment interview at the RMIT Bundoora West campus or City campus, you may ask any questions you may have concerning this study to help you make the final decision to participate. You will be asked to sign an informed consent form before taking further examinations. During this interview, you are required to complete an assessment questionnaire, undertake an allergy test (skin prick test) and have a nasal examination. If you meet the inclusion criteria, you will be given assessment forms (including 7-point scale symptom severity assessment questionnaire, rhinoconjunctivitis quality of life questionnaire with standardized activities [RQLQs], and records of medication usage for your hay fever) to complete during the following two-week run-in period. You are not allowed to have any acupuncture or acupressure treatment from the initial interview to the endpoint of the trial (14 weeks).

Your participation will involve an initial assessment interview, a two-week run-in period, a four-week (specific or non-specific) treatment period and an eight-week follow-up period. In total your involvement in this research project will continue for 14 weeks.

You will be asked to come to the Clinical Trial Laboratory every week during the four-week treatment period. During the first visit, you need to bring the completed forms back. You will also be asked to randomly pick an envelope which contains randomisation code and pass it onto a registered acupuncturist (Ms Yaqun Liang). The registered acupuncturist (Ms Yaqun Liang) will provide detailed instructions to you on how to perform acupressure by yourself. You will be given a sheet illustrating the location of five acupuncture points. You will be asked to perform self-administered acupressure on those five acupuncture points each time, one minute each point, twice per day.

In the second, third and fourth visits, participants will return the completed forms for the previous week and receive a new set of questionnaires for completion. The registered acupuncturist (Ms Yaqun Liang) will reinforce the participants' techniques on self-administered acupressure.

You will also be asked to complete the following forms every week:

- - 7-point scale symptom severity assessment;
  - rhinoconjunctivitis quality of life questionnaire with standardized activities (RQLQs);
  - medication usage form;
  - acupressure dosage form; and
  - adverse events form (unexpected feelings related to self-administered acupressure).

In the first and the final week of the treatment period, you will be required to record your opinions about the self-administered acupressure.

During the eight-week follow-up period, you will be asked to stop performing self-administered acupressure and complete the following forms fortnightly:

- - 7-point scale symptom severity assessment;
  - rhinoconjunctivitis quality of life questionnaire with standardized activities (RQLQs);
  - medication usage form; and
  - adverse events form (unexpected feelings related to self-administered acupressure).

Completing all the questionnaires each time will take approximately 10 minutes. You may submit all the completed assessment forms electronically (ie. scan or take photo of all forms via email). All the data will be stored in the password-protected computer at RMIT University.

There are no costs associated with participating in this research project, nor will you be paid.

**4 Other relevant information about the research project**

This project is to conduct randomised, controlled clinical trials designed by the Discipline of Chinese Medicine, RMIT University. You will be joining more than 110 other hay fever sufferers who come from communities in Melbourne. You will have 50% chance of being randomly assigned to either specific acupressure group or non-specific acupressure group. This design allows us to compare the therapeutic effects of the specific treatment with the non-specific control treatment. The specific treatment is to apply self-administered acupressure on five acupuncture points which have been demonstrated to have specific effects on hay fever; on the other hand, non-specific treatment refers to applying self-administered acupressure on five acupuncture points which do not have specific effects on hay fever according to the literature.

**5 Do I have to take part in this research project?**

Participation in any research project is voluntary. If you do not wish to take part, you do not have to. If you decide to take part and later change your mind, you are free to withdraw from the project at any stage.

Those will not affect your relationship with the researchers or with RMIT University.

If you do decide to take part, you will be given this Participant Information Sheet and Consent Form to sign and you will be given a copy to keep. Submitting your completed questionnaires is an indication of your consent to participate in the study.

**6 What are the possible benefits of taking part?**

We cannot guarantee or promise that you will receive any benefits from this research; however, you may be contributing to knowledge advancement. Possible benefits may include that you will be within the first group of subjects to learn about self-administered acupressure to improve your health. If the study has significant results, it will benefit all people suffering from hay fever. This may help hay fever sufferers to manage their conditions using self-administered acupressure, as well as reducing medication usage, visit times, and financial cost.

**7 What are the risks and disadvantages of taking part?**

All the acupoints to be used in this research project are well documented in the literature and no adverse events have been reported associated with the selected acupoints. During self-administered acupressure, you may feel soreness within the acupoint regions. This is common and normal feeling of acupressure. Self-administered acupressure has minimal risks and is non-invasive. Pain or discomfort associated with acupressure is very rare. However, if you have any concerns, please contact the researchers or you may be referred to a local GP.

Self-administered acupressure does not cause any direct psychological distress. However if you become upset or distressed as a result of your participation in the research project, members of the research team will discuss appropriate support for you.

The discomfort of skin prick test is small and the systemic reactions are minimal. A large survey has showed that the rate of systemic allergic reactions caused by skin prick test was 0.033%, all occurring in asthmatics.

The allergen skin prick test will be performed by a trained practitioner and a general practitioner (GP) will be available on site. If the participant experiences itchiness after 15 minutes, topical creams (such as urea creams) or an ice-pack will be applied to reduce itching. If an anaphylactic reaction occurs in a rare circumstance, the investigators will follow the minimum standards suggested by the Australasian Society of Clinical Immunology and Allergy for management, provide the followings and call ambulance:

- oxygen, 6l/min via mask;
- adrenaline for intramuscular injection;
- intravenous cannulation and intravenous fluids for rapid infusion in case of hypotension;
- salbutamol via nebuliser or spacer.

You can contact any investigator of this project for further discussion:

- Dr Angela Yang: 03 9925 7175; email: [angela.yang@rmit.edu.au](mailto:angela.yang@rmit.edu.au)
- Ms Yaqun Liang: 03 9925 7584; email: [s3220226@student.rmit.edu.au](mailto:s3220226@student.rmit.edu.au)
- Dr George Lenon: 03 9925 6587; email: [george.lenon@rmit.edu.au](mailto:george.lenon@rmit.edu.au)

**8 What if I withdraw from this research project?**

If you decide to withdraw from the project, please notify a member of the research team. However, your data will not be destroyed and will be used for analysis.

**9 What happens when the research project ends?**

When the research project is completed, participants will be provided with a plain language summary of the findings of the study.

Findings of the study will also be presented to scientific conference and submitted to scientific journals for publication. However, only synthesised data will be published in the results, no name or other forms of participants’ identification will be reported. All information in this project will not be disclosed, nor can it be accessed.

Upon completion of the research project, participants in non-specific acupressure group will be informed and instructed with the specific acupressure treatment.

**How is the research project being conducted?**

**10 What will happen to information about me?**

By signing the consent form you consent to the research team collecting and using information from you for the research project. Any information obtained in connection with this research project that can identify you will remain confidential. All information provided by you and the data collected through this research project will be stored in a password protected university’s computer. All participants’ files will be kept in a locked cabinet during the trial and stored in storage room and will be retained for 15 years and then will be shredded and disposed as required by the National Health and Medical Research Council (NHMRC) privacy policy. Only the individuals listed in this application will have the access to the information. Your records may be inspected only by authorised persons participating in the research for the purpose of an original data audit.

It is anticipated that the results of this research project will be published and/or presented in a variety of forums. In any publication and/or presentation, all your personal information will be removed so your identity will not be revealed.

In accordance with relevant Australian and/or Victorian privacy and other relevant laws, you have the right to request access to the information about you that is collected and stored by the research team. You also have the right to request that any information with which you disagree be corrected. Please inform the research team member named at the end of this document if you would like to access your information.

Any information that you provide can be disclosed only if (1) it is to protect you or others from harm, (2) if specifically allowed by law, (3) you provide the researchers with written permission. Any information obtained for the purpose of this research project that can identify you will be treated as confidential and securely stored.

**11 Who is organising and funding the research?**

This research project is being conducted by Dr Angela Wei Hong Yang, Dr George Binh Lenon and Ms Yaqun Liang. The School of Health and Biomedical Sciences at RMIT University supports and funds this research.

**12 Who has reviewed the research project?**

All research in Australia involving humans is reviewed by an independent group of people called a Human Research Ethics Committee (HREC). This research project has been approved by the RMIT University HREC.

This project will be carried out according to the *National Statement on Ethical Conduct in Human Research* (2015). This statement has been developed to protect the interests of people who agree to participate in human research studies.

**13 Further information and who to contact**

If you want any further information concerning this project, you can contact any of the following people:

**Research contact person**

| Name | Yaqun Liang |
| --- | --- |
| Position | Research student |
| Telephone | 61 3 9925 7584 |
| Email | s3220226@student.rmit.edu.au |

**Research contact person**

| Name | Angela Wei Hong Yang |
| --- | --- |
| Position | Chief investigator / Senior supervisor |
| Telephone | 61 3 9925 7175 |
| Email | [angela.yang@rmit.edu.au](mailto:angela.yang@rmit.edu.au) |

**Research contact person**

| Name | George Binh Lenon |
| --- | --- |
| Position | Associate investigator / Associate supervisor |
| Telephone | 61 3 9925 6587 |
| Email | [george.lenon@rmit.edu.au](mailto:george.lenon@rmit.edu.au) |

**14 Complaints**

Should you have any concerns or questions about this research project, which you do not wish to discuss with the researchers listed in this document, then you may contact:

| Reviewing HREC name | RMIT University |
| --- | --- |
| HREC Secretary | Peter Burke |
| Telephone | 03 9925 2251 |
| Email | [human.ethics@rmit.edu.au](mailto:human.ethics@rmit.edu.au) |
| Mailing address | Research Ethics Co-ordinator  Research Integrity Governance and Systems  RMIT University  GPO Box 2476  MELBOURNE VIC 3001 |

**Consent Form**

| **Title** | Self-administered acupressure for the management of allergic rhinitis: Randomised controlled trials |
| --- | --- |
| **Chief Investigator/Senior Supervisor** | Dr Angela Wei Hong Yang |
| **Associate Investigator(s)/Associate Supervisors** | Dr George Binh Lenon |
| **Research Student(s)** | Ms Yaqun Liang |
|  |  |

**Acknowledgement by Participant**

I have read and understood the Participant Information Sheet.

I understand the purposes, procedures and risks of the research described in the project.

I have had an opportunity to ask questions and I am satisfied with the answers I have received.

I freely agree to participate in this research project as described and understand that I am free to withdraw at any time during the project without affecting my relationship with RMIT.

I understand that I will be given a signed copy of this document to keep.

|  | | | | | | | |
| --- | --- | --- | --- | --- | --- | --- | --- |
|  | Name of Participant (please print) | |  | |  |  |  |
|  | | | | | | | |
|  | Signature |  | | Date | |  |  |
|  | | | | | | | |

**Declaration by Researcher^†^**

I have given a verbal explanation of the research project, its procedures and risks and I believe that the participant has understood that explanation.

|  | | | | | | |
| --- | --- | --- | --- | --- | --- | --- |
|  | Name of Researcher^†^ (please print) | |  | | |  |
|  | | | | | |  |
|  | Signature |  | | Date |  |  |
|  | | | | | | |

^†^ An appropriately qualified member of the research team must provide the explanation of, and information concerning, the research project.

Note: All parties signing the consent section must date their own signature.
